# Multi-Omics Signatures Identification for LUAD Prognosis Prediction Model Based on the Integrative Analysis of Immune and Hypoxia Signals

**DOI:** 10.3389/fcell.2022.840466

**Published:** 2022-03-10

**Authors:** Yuqing Lou, Qin Shi, Yanwei Zhang, Ying Qi, Wei Zhang, Huimin Wang, Jun Lu, Baohui Han, Hua Zhong

**Affiliations:** ^1^ Department of Pulmonary Medicine, Shanghai Chest Hospital, Shanghai Jiao Tong University, Shanghai, China; ^2^ Department of Oncology, Baoshan Branch of Shuguang Hospital, Shanghai University of Traditional Chinese Medicine, Shanghai, China; ^3^ School of Basic Medical Science, Hangzhou Normal University, Hangzhou, China; ^4^ Shanghai Institute of Thoracic Oncology, Shanghai Chest Hospital, Shanghai Jiao Tong University, Shanghai, China; ^5^ Translational Medical Research Platform for Thoracic Oncology, Shanghai Chest Hospital, Shanghai Jiao Tong University, Shanghai, China; ^6^ Department of Bio-bank, Shanghai Chest Hospital, Shanghai Jiao Tong University, Shanghai, China

**Keywords:** lung adenocarcinoma, multi-omics biomarker, immune, hypoxia, prognosis prediction

## Abstract

Lung adenocarcinoma (LUAD) is the most common histological subtype of lung cancer with heterogeneous outcomes and diverse therapeutic responses. However, the understanding of the potential mechanism behind LUAD initiation and progression remains limited. Increasing evidence shows the clinical significance of the interaction between immune and hypoxia in tumor microenvironment. To mine reliable prognostic signatures related to both immune and hypoxia and provide a more comprehensive landscape of the hypoxia-immune genome map, we investigated the hypoxia-immune-related alteration at the multi-omics level (gene expression, somatic mutation, and DNA methylation). Multiple strategies including lasso regression and multivariate Cox proportional hazards regression were used to screen the signatures with clinical significance and establish an incorporated prognosis prediction model with robust discriminative power on survival status on both the training and test datasets. Finally, combing all the samples, we constructed a robust model comprising 19 signatures for the prognosis prediction of LUAD patients. The results of our study provide a comprehensive landscape of hypoxia-immune related genetic alterations and provide a robust prognosis predictor for LUAD patients.

## Introduction

Lung cancer is one of the most common and severe types of cancer and presents the leading cause of both incidence and mortality worldwide in both genders (Siegel et al., 2019; Lu et al., 2021a; Lu et al., 2021b; Chu et al., 2021). Lung adenocarcinoma (LUAD) is the most common histological subtype of lung cancer with an increasing incidence over the past few decades (Ferlay et al., 2010; Lou et al., 2020; Lou et al., 2021). Although advances on treatment strategies for LUAD has been made, the overall 5-year survival rate is still at a low level with unoptimistic prognosis (less than 20%) (Wu et al., 2021). Although the routine display of clinicopathologic features by WHO classification and TNM staging system is important for the selection of appropriate treatment (Barth, 2020), these approaches appear to be inadequate due to the heterogeneity among patients.

The tumor microenvironment (TME), consisting of tumor cells, endothelial cells, immune cells, fibroblasts, macrophages, and the extracellular matrix, is a key regulator of carcinogenesis that substantially influences the initiation, development, and progression of LUAD, as well as the response to various therapy approaches (Anderson and Simon, 2020; Fu et al., 2021). Different components of the TME can regulate the development and progression of tumor. The immune cells, which are called tumor-infiltrating lymphocytes or TILs, can detect and destroy abnormal cells and potentially prevent or curb the tumor growth (Lei et al., 2020; Hajiran et al., 2021). In immune cells, reactive oxygen species (ROS) are mediators of several pivotal functions (e.g., phagocytosis, antigen presentation and recognition, cytolysis, as well as phenotypical differentiation) and exert immunosuppressive effects on T and natural killer (NK) cells (Kennel and Greten, 2021). Most recently, hypoxia has been reported as an intrinsic characteristic of solid tumor and plays an important role in cancer progression, angiogenesis, metastasis, and resistance to therapy (Muz et al., 2015). In the tumor microenvironment, the blood vessels and fibroblasts can influence the perfusion and diffusion of O2, leading to the development of hypoxia in the tissue region (Belcher et al., 2020). Beyond the vasoconstriction, hypoxia recruits bone marrow precursor cells to the lung and affects the behavior of immune cells (Nicolls and Voelkel, 2007). Moreover, hypoxia is one of the reasons for poor therapy efficacy of current anti-angiogenic drugs and was reported to be associated with resistance to PD-1 blockade in squamous cell carcinoma of the head and neck (Jiang et al., 2020; Zandberg et al., 2021). Salem et al. (2018) outlined past and ongoing hypoxia-targeted therapy trials in NSCLC and highlight the potential of hypoxia as a therapeutic target. Therefore, further study on the relationship between hypoxia and immunity in LUAD is required to develop new therapeutic strategies.

In this study, we hypothesized that immune and hypoxia interaction may provide prognostic value in LUAD patients. Based on the expression profiles from The Cancer Genome Atlas (TCGA) portal, we aim to identify the hypoxia and immune status for each sample using the expression of pan-cancer metagenes for 28 immune cell subpopulations and the hypoxia related genes, respectively. Then, we will correlate the hypoxia-immune status with multi-omics genetic alterations to screen the hypoxia-immune biomarkers and finally establish an incorporated prognosis prediction model. The results of this study are expected to provide a more comprehensive hypoxia-immune genome map and may provide a better prognosis predictor for LUAD patients.

## Results

### Immune Status and Immune-Related DEGs in LUAD

**FIGURE 1:**
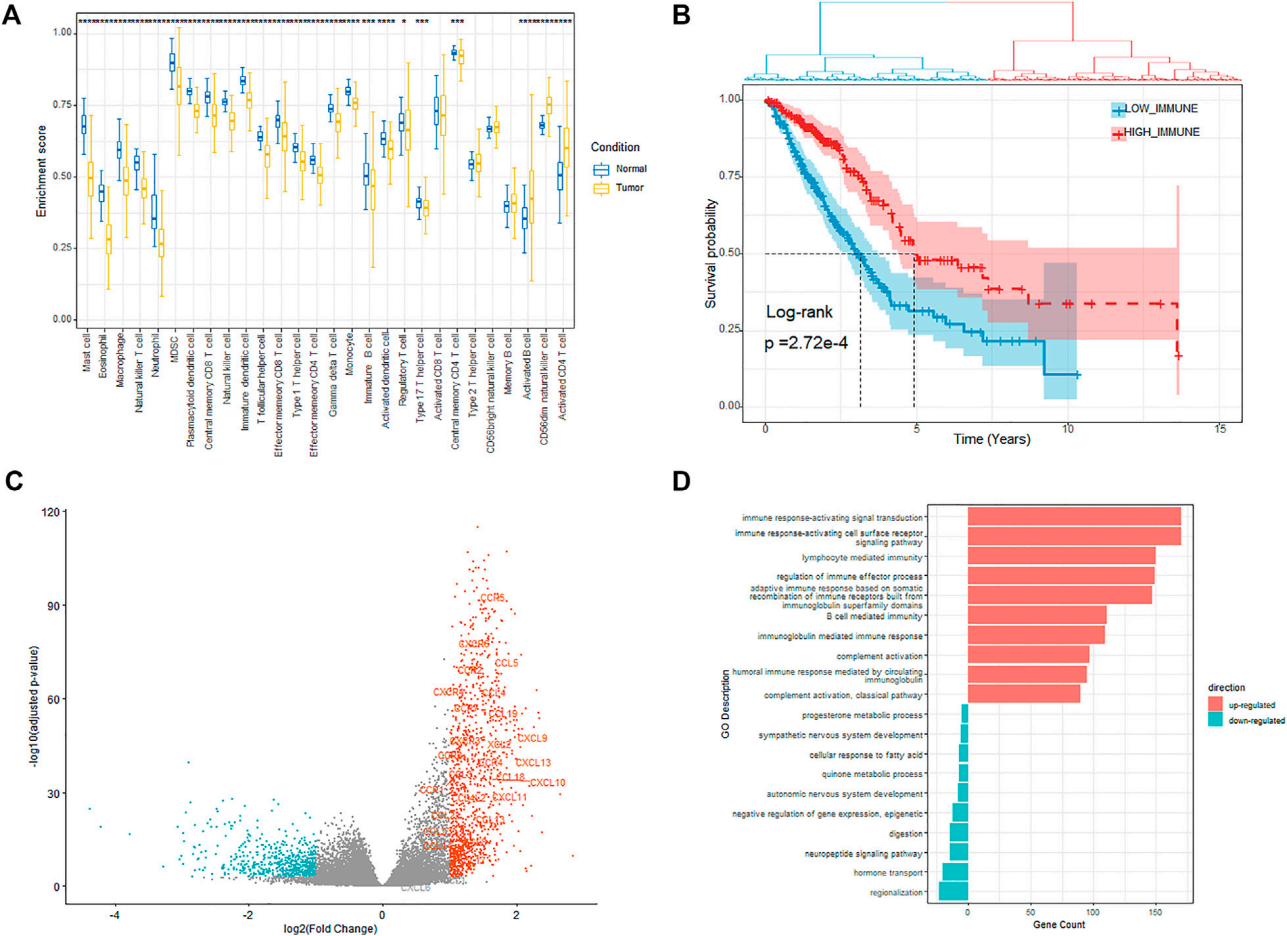
Investigation of the immune status. **(A)** Enrichment of different immune cells between tumor and normal samples. **(B)** Kaplan–Meier plot of overall survival for patients regarded as high- and low immunity. **(C)** Volcano plot showing the differentially upregulated (red points) and downregulated genes (blue points). **(D)** Bar plot showing the top 10 enrichment of biological processes (GOBP) for the up-regulated and down-regulated genes respectively in the high-immunity cohort.

Based on the immune-related genes (IRGs) for 28 immune cell subpopulations provided in Charoentong et al. (2017) study, we calculated the enrichment scores (ESs) for each of the 569 samples (including 510 tumor samples and 58 normal samples) using the RNA-seq profile by Gene Set Variation Analysis (GSEA) (Hänzelmann et al., 2013). The results showed that the ESs of 25 in 28 immune cells members were significantly different between the tumor and normal samples. Most of the immune cell members were significantly enriched in the normal samples rather than in tumor samples, except the activate B cell, CD56^dim^ natural killer cell, and activate CD4 T cell (Figure 1A). The tumor-infiltrating B cells (TIBs) play a multifaceted dual role in regulating tumor immunity rather than just tumor inhibition or promotion and affect the function of other immune cells such as CD4^+^ T cells and natural killer cells in the tumor microenvironment (Guo and Cui, 2019). We also observed that the enrichment of several immune cell members was also significantly different among different tumor stages (Supplementary Figure S1). Based on the ESs profile of the 28 types of immune cells in all the tumor samples, we defined the immune status for the 510 primary tumor samples and divided the related LUAD patients into two groups using hierarchical clustering with “ward.D” agglomeration method, which aims to find compact, spherical clusters by selecting clusters to merge based on the change in the cluster variances (Figure 1B), yielding 215 and 295 patients in the two groups, respectively. Survival comparison showed significant differences among the two groups (HR = 0.566, *p*-value = 2.72e-4), and the groups with better prognosis were labeled as IMMUNITY_H and others as IMMUNITY_L.

We next explored the expression alteration between the IMMUNITY_H and IMMUNITY_L cohorts to identify the immune-related DEGs. The genes with fold change larger than 2 and FDR less than 0.001 were regarded as differentially expressed, of which 1,118 and 628 genes were, respectively, up-regulated and down-regulated in the IMMUNITY_H cohort (Figure 1C). From the results, we observed that most of the chemotactic factors (e.g., CCR5, CXCR6, and CCL5), which are pivotal mediators of host defense and orchestrate the recruitment of immune cells into sites of infection and inflammation, were significantly up-regulated in the IMMUNITY_H samples.

The functional enrichment analyses of the up-regulated and down-regulated genes were performed using clusterProfile package (Yu et al., 2012). The results showed that the up-regulated genes were enriched in immune-related biological processes such as T cell activation and leukocyte proliferation, which indicated that the up-regulated genes play a positive role in the enhancement of tumor-associated immunity (Figure 1D, Supplementary Table S1). On the other hand, the down-regulated genes were mainly enriched in nervous system development related biological processes, which indicated that some downregulated genes modulate the activities of immune and tumor cells through affecting nervous system development (Supplementary Table S1). For example, PHOX2A/B, which is a paired-like homeodomain transcription factor that participates in specifying the autonomic nervous system, was verified as a tumor suppressor (Wilzén et al., 2009; Pudela et al., 2020). The KEGG pathway enrichment analysis results also showed that the up-regulated genes were mainly enriched in immune-related pathways, while the down-regulated genes were enriched in the pathways related with nervous system development and metabolism (Supplementary Table S1).

### Identification of Hypoxia-Immune–Related Subtype and Associated Prognostic DEGs

**FIGURE 2:**
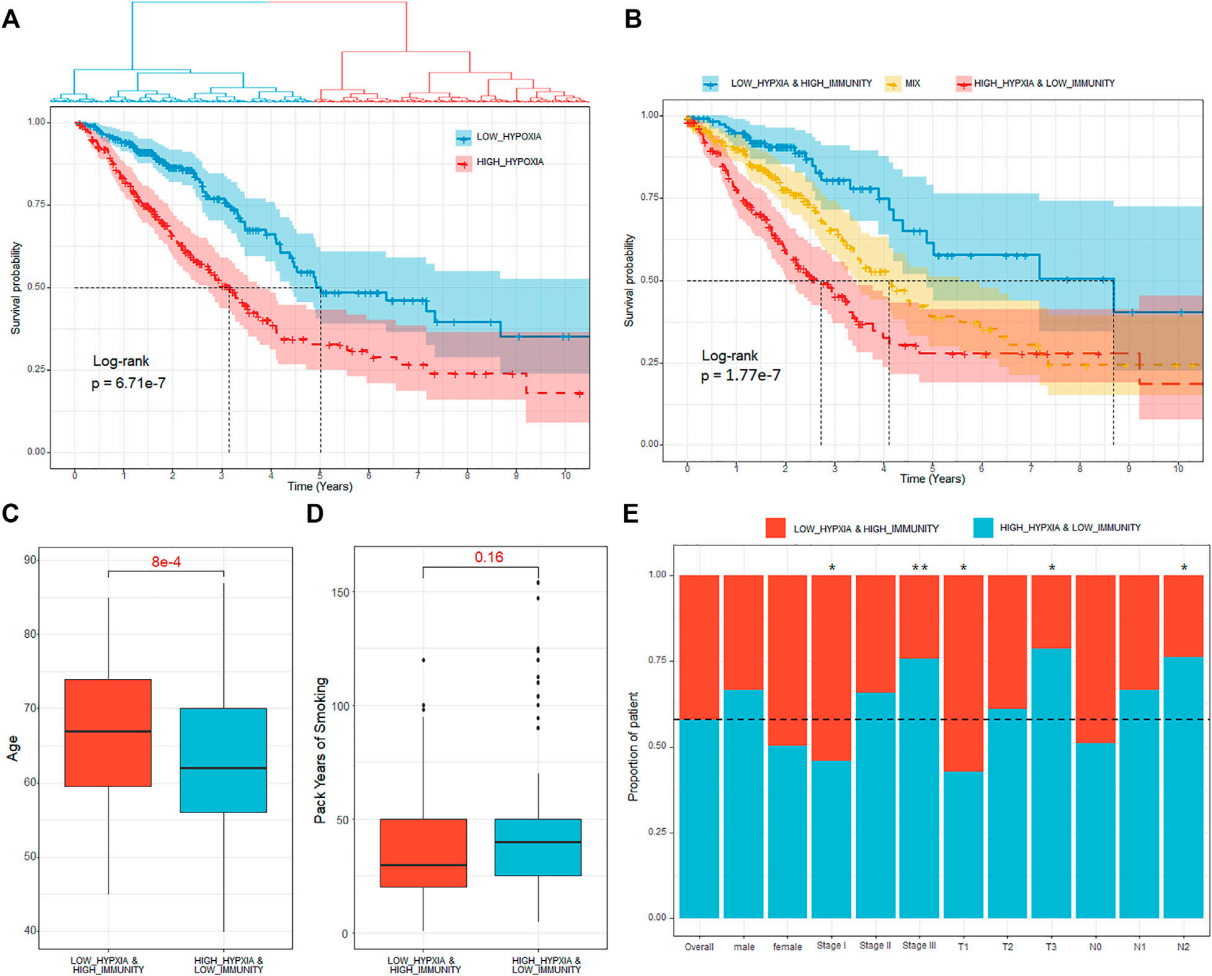
Definition of hypoxia-immune–related subtypes. **(A)** Kaplan–Meier plot of overall survival for patients regarded as high- and low hypoxia. **(B)** Kaplan–Meier plot of overall survival for the “HYPOXIA_L & IMMUNITY_H,” “MIX,” and “HYPOXIA_H & IMMUNITY_L” cohorts. **(C)** Age comparison of patients in different cohorts. The *p*-value were calculated using Wilcoxon test. **(D)** Pack of years of smoke comparison of patients in different cohorts. The *p*-value were calculated using Wilcoxon test. **(E)** Proportion of patients in “HYPOXIA_L & IMMUNITY_H” and “HYPOXIA_H & IMMUNITY_L” cohorts respect to various clinical factors. Fisher’s exact test is used to measure the significance. * means the correlation *p*-value is less than 0.05, ** means the correlation *p*-value is less than 0.01.

To deduce the hypoxia status for each of the samples, we extracted the expression of the 200 hypoxia-related hallmark genes and then handled with UMAP (Uniform Manifold Approximation and Projection). Using the latent variables generated by UMAP, we further divided the patients into two groups (Figure 2A, *Methods and Materials*). There were 249 and 261 patients in the two groups and the survival analysis showed significant difference between the two groups (Figure 2A, HR = 2.15, *p*-value = 6.71e-7). The patients with better prognosis were assigned to HYPOXIA_L group and others to HYPOXIA_H group. Taking the immune and hypoxia statuses together, we divided the patients into three groups, which are “HYPOXIA_L & IMMUNITY_H” (*n* = 124), “HYPOXIA_H & IMMUNITY_L” (*n* = 170), and “MIX” (*n* = 216). The survival analysis results revealed that the OS times of patients in different groups were significantly different (Figure 2B, HR = 3.33, *p*-value = 1.77e-7), and the “HYPOXIA_L & IMMUNITY_H” cohort harbored the best prognosis, while the patients in the “HYPOXIA_H & IMMUNITY_L” yield the worst prognosis.

We further investigated the dispersion of various clinical characters (e.g., age, clinical stage, tumor size, lymph node, and distant metastasis) between the cohorts with different hypoxia-immune status. Through Cox proportional hazards regression analysis, we observed that the OS time is independent from age (HR = 1, *p*-value = 0.64). However, we observed that the patients regarded as “HYPOXIA_H & IMMUNITY_L” were significantly younger than patients in the “HYPOXIA_L & IMMUNITY_H” group (Figure 2C, Wilcox test *p*-value = 8e-4), which may explain the clinical observation that young lung patients tend to present with advanced disease at diagnosis, resulting in an extremely poor survival (Rocha et al., 1994). Besides that, we also observed that the patients with more packs years of smoke tend to enriched in the high-risk (“HYPOXIA_H & IMMUNITY_L”) cohort (Figure 2D). Moreover, we also pay attention to association between the immune-hypoxia status and various clinical factors, such as gender and clinical stages. Generally, gender was independent from the immune-hypoxia status (Figure 2E). For the clinical stage, we observed that the patients in the stage I tended to be in the “HYPOXIA_L & IMMUNITY_H” cohort (Fisher’s exact test, *p*-value = 0.02, Figure 2E), while the patients in the stage III tended to be in the “HYPOXIA_H & IMMUNITY_L” cohort (Fisher’s exact test, *p*-value = 0.007, Figure 2E). As the distant metastases were present in only 4.48% of the patients selected for comparison, we only consider the “N” (Regional lymph nodes) and “T” (Primary tumor) for the TNM dispersion analysis. The results showed that the patients with higher tumor size were significantly enriched in the “HYPOXIA_H & IMMUNITY_L” group, and the patients with more lymph nodes that contain cancer were also significantly enriched in the “HYPOXIA_H & IMMUNITY_L” group. These results further indicated that the patients in the “HYPOXIA_H & IMMUNITY_L” cohort tend to be high-risk.

The hypoxia-immune-related DEGs were obtained by comparing the expression between the “HYPOXIA_L & IMMUNITY_H” and “HYPOXIA_H & IMMUNITY_L” cohorts, and finally 2,798 DEGs were obtained with fold-change larger than two and adjusted *p*-value less than 0.001 (Supplementary Table S2). The 1,091 genes significantly up-regulated in the “HYPOXIA_H & IMMUNITY_L” cohort where patients yielded worse survival were regarded as risk DEGs (e.g., *GAPDH*, *NTS, LDHA,* and *CDH2*), and the 1,707 genes significantly up-regulated in the “HYPOXIA_L & IMMUNITY_H” cohort where patients yielded better outcome were regarded as protective DEGs (e.g., *RCSD1, IL16, PRB4,* and *VEGFD*).

### Comparing Somatic Mutations Between Different Hypoxia-Immune Status

After identifying the gene signatures associated with the hypoxia-immune status, we also tended to explore the alteration at genome level between the “HYPOXIA_L & IMMUNITY_H” and “HYPOXIA_H & IMMUNITY_L” cohorts. The varscan2 results about single-nucleotide variant (SNV), single-nucleotide polymorphism (SNP), insertion (INS), and deletion (DEL) were used in this part. We observed that majority of the genomic variants were missense mutation in both the “HYPOXIA_L & IMMUNITY_H” and “HYPOXIA_H & IMMUNITY_L” cohorts (around 85%), while for most types, the samples in the “HYPOXIA_H & IMMUNITY_L” cohort harbored a significantly larger number of variants than those in the “HYPOXIA_L & IMMUNITY_H” (Supplementary Table S3, Supplementary Figure S2). The ratios between transversion (Tv) and transition (Ti) in all SNVs were approximately 2:1 and remained stable in both cohorts. Moreover, we also observed that the TMB of the patients in the “HYPOXIA_H & IMMUNITY_L” cohort was significantly larger than that of patients in the “HYPOXIA_L & IMMUNITY_H” (Wilcox test *p*-value = 1.47e-7), which also indicated that the “HYPOXIA_H & IMMUNITY_L” is high-risk status.

**FIGURE 3:**
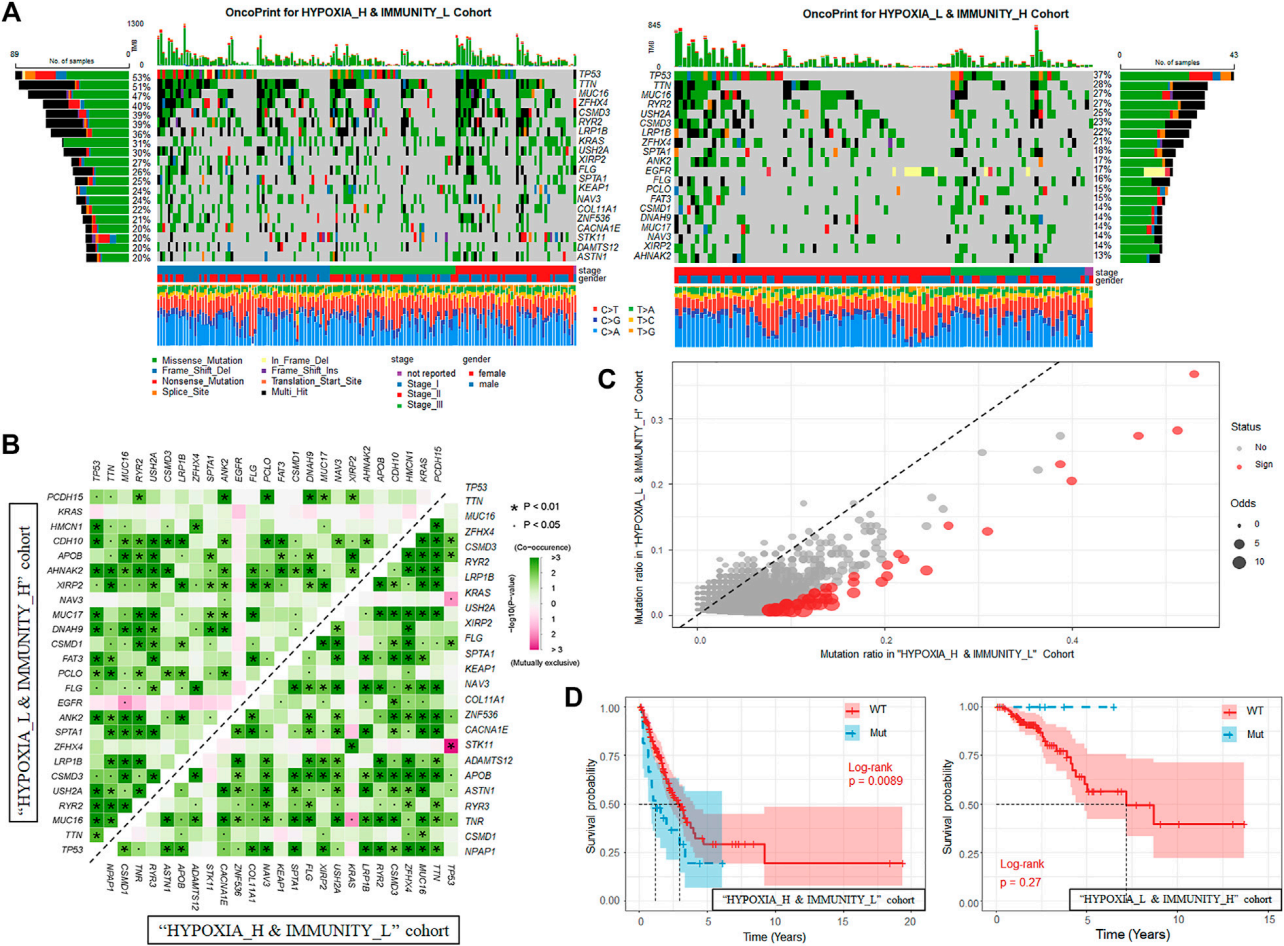
Landscape of Somatic Mutation in “HYPOXIA_L & IMMUNITY_H” and “HYPOXIA_H & IMMUNITY_L” cohorts. **(A)** Waterfall plot shows the mutation distribution of the top 20 most frequently mutated genes. The central panel shows the types of mutations in each LUAD sample. The upper panel shows the mutation frequency of each LUAD sample. The bar plots on the left and right side show the frequency and mutation type of genes mutated in the “HYPOXIA_H & IMMUNITY_L” and “HYPOXIA_L & IMMUNITY_H” cohorts, respectively. The lower part shows the clinical features (tumor stage and sex) and SNV types of each sample. The bottom panel is the legend for mutation types and clinical features. **(B)** The mutually co-occurring and exclusive mutations of the top 25 frequently mutated genes in “HYPOXIA_H & IMMUNITY_L” and “HYPOXIA_L & IMMUNITY_H” cohorts, respectively. The color and symbol in each cell indicated the statistical significance of the association for each pair of genes. **(C)** Scatter plot of differentially mutated genes between the “HYPOXIA_H & IMMUNITY_L” and “HYPOXIA_L & IMMUNITY_H” cohorts. Fisher’s test was used to measure the statistical significance and genes with *p*-value less than 0.01 were regarded significantly mutated. **(D)** Kaplan-Meier curves show the independent relevance between overall survival time and *CRB1* mutation in “HYPOXIA_H & IMMUNITY_L” and “HYPOXIA_L & IMMUNITY_H” cohorts, respectively.

In the “HYPOXIA_H & IMMUNITY_L” cohort, 181 genes were mutated in more than 10% of the samples while only 44 genes met this criterion in the “HYPOXIA_L & IMMUNITY_H” cohort, of which there was an overlap of 42 genes. The top 20 most frequently mutated genes in the corresponding cohorts were shown in Figure 3A. From the results, we observed that TP53, TTN, and MUC16 rank among the top 3 most frequently mutated genes in the corresponding cohorts. These genes were reported to be interactive and regulated various tumor associated biological processes (Huang et al., 2021; Wu et al., 2021; Zhao et al., 2021; Zhou et al., 2021). We next investigated the co-occurring and exclusive mutation of the top 25 frequently mutated genes (Figure 3B). Compared with the pervasive co-occurrence landscape (280 cases), there were only four unique cases in the two cohorts (KRAS-TP53: *p*-value = 0.013, KRAS-TNR: *p*-value = 0.011, TP53-STK11: *p*-value = 1.87e-4, and MUC16-EGFR: *p*-value = 0.013) exhibiting mutually exclusive mutations, which suggests their probably redundant effect in the same pathway and selective advantages between them to keep more than one copy of the mutations. To extract the signatures at the somatic genome level, we applied Fisher’s test to identify the differentially mutated genes between the two cohorts, and finally 54 genes were regarded as significantly differentially mutated (*p*-value < 0.01, Figure 3C). From the results, we found that the genes mutated more frequently in the “HYPOXIA_H & IMMUNITY_L” cohort than in the “HYPOXIA_L & IMMUNITY_H” cohort. To verify the same mutation may exert distinct impacts on the survival time of patients grouped in different cohorts, we divided the patients in both the “HYPOXIA_L & IMMUNITY_H” and “HYPOXIA_H & IMMUNITY_L” cohorts into “wt” and “mut” groups. The survival analysis results showed that several genes can divide the patients into two groups with significantly different OS times in one cohort, while they cannot in the other cohort (Supplementary Table S4). For example, the OS times of the patients in the “HYPOXIA_H & IMMUNITY_L” cohorts with and without *CRB1* mutation were significantly different (HR = 3.09, *p*-value = 5.11e-6), while no such significant difference was observed in “HYPOXIA_L & IMMUNITY_H” (Figure 3D). And TPR showed the opposite result (Figure 3D).

### Comparing DNA Methylation Level Between Different Hypoxia-Immune Status

**FIGURE 4:**
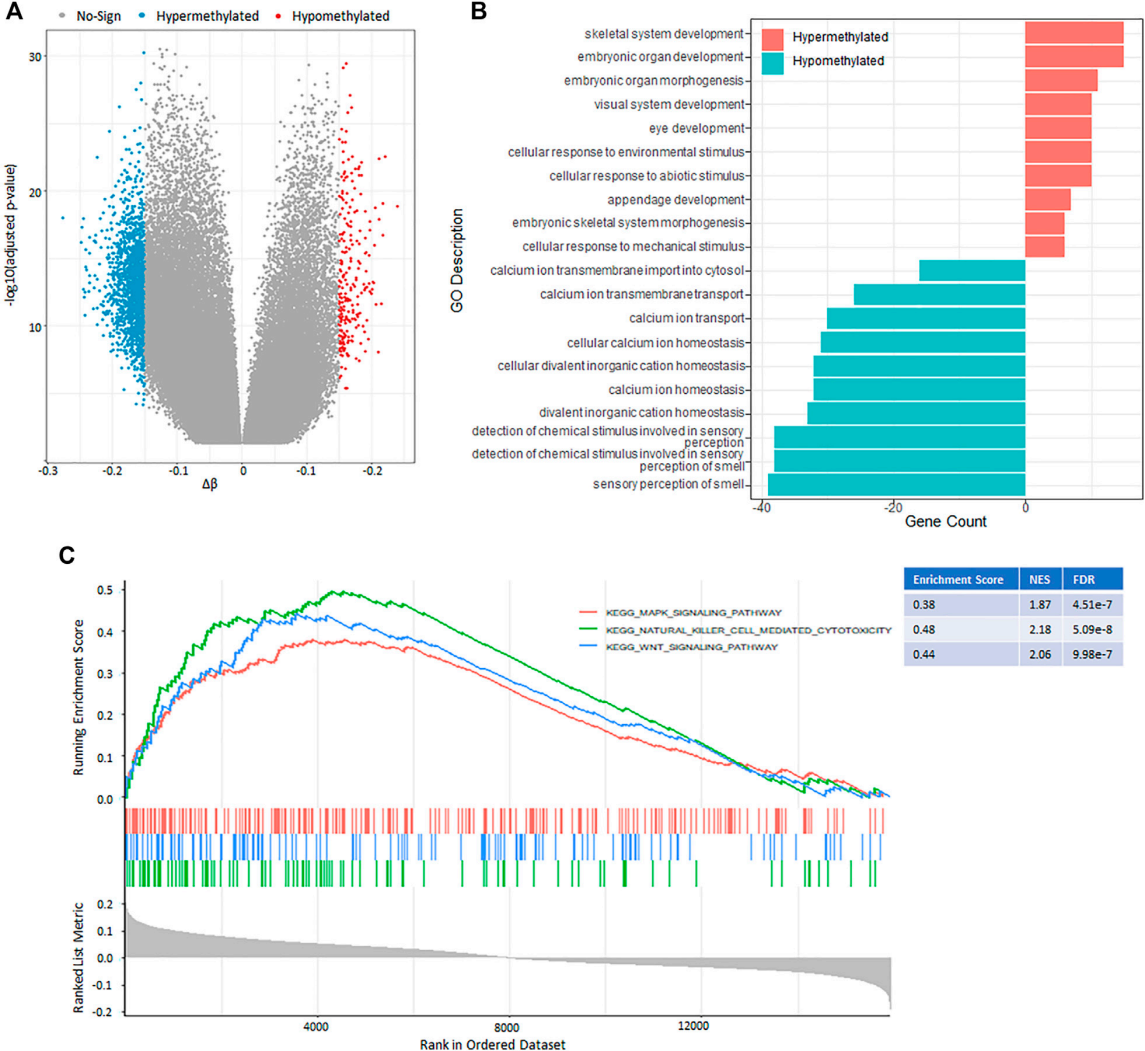
DNA methylation pattern between the “HYPOXIA_L & IMMUNITY_H” and “HYPOXIA_H & IMMUNITY_L” cohorts. **(A)** Volcano plot of the genome-wide DNA differential methylation between the two cohorts. **(B)** Bar plot showing the top 10 enrichment of biological processes (GOBP) for the hypermethylated and hypomethylated genes, respectively in the “HYPOXIA_H & IMMUNITY_L” cohort. **(C)** GSEA results show the significant enrichment in three cancer related pathways. Genes were ranked by Δβ.

Aberrations in DNA methylation system have an important role in human disease, and DNA methylation patterns are globally disrupted in cancer, with genome-wide hypomethylation and gene-specific hypermethylation events occurring simultaneously in the same cell (Robertson, 2005). In this section, we aimed to identify and compare the effects of DNA methylation patterns in different hypoxia-immune cohorts using Illumina Infinium 450k DNA methylation data. Only the patients grouped into “HYPOXIA_L & IMMUNITY_H” or “HYPOXIA_H & IMMUNITY_L” cohorts were considered. After preprocessing, 264 samples in which no more than 20% probes have missing beta values were used to detect the differential methylation probes (DMPs) using ChAMP(Morris et al., 2014; Tian et al., 2017) (see *Methods and Materials*). Finally, 2,082 hypoxia-immune-related DMPs were identified with the criterion of absolute Δβ larger than 0.15 and adjusted *p*-value less than 0.05 (Figure 4A, Supplementary Table S5). Compared with “HYPOXIA_L & IMMUNITY_H” cohort, 1844 (88.57%) hypomethylated positions involving 520 genes were identified in the “HYPOXIA_H & IMMUNITY_L” cohort, while only 238 (11.43%) positions related to 128 genes were significantly hypermethylated. These results indicated that the “HYPOXIA_H & IMMUNITY_L” cohort tends to have hypomethylated positions overall. Only 3 genes (*ZC3H12D*, *XKR6*, *DIP2C*) contain both hypermethylated and hypomethylated positions. Among these 520 hypomethylated genes in the “HYPOXIA_H & IMMUNITY_L” cohort, 29 and 23 genes were significantly upregulated and downregulated, respectively. In contract, there were only 4 upregulated and 5 downregulated genes among the hypermethylated genes.

The functional enrichment analysis results revealed that the hypomethylated genes mainly involved in sensory perception, ion transport, and ion homeostasis, while the hypermethylated genes play potential roles in development and cellular response (Figure 4B, Supplementary Table S6). The gene set enrichment analysis (GSEA) of these DMP-associated genes showed that hypermethylated genes with highly positive beta difference have more essential contributions to various cancer related pathways such as natural killer cell mediated cytotoxicity, Wnt signal pathway, and Mapk signal pathway (Figure 4C, Supplementary Table S7).

### Prognostic Prediction Using Multi-Omics Signatures

**FIGURE 5:**
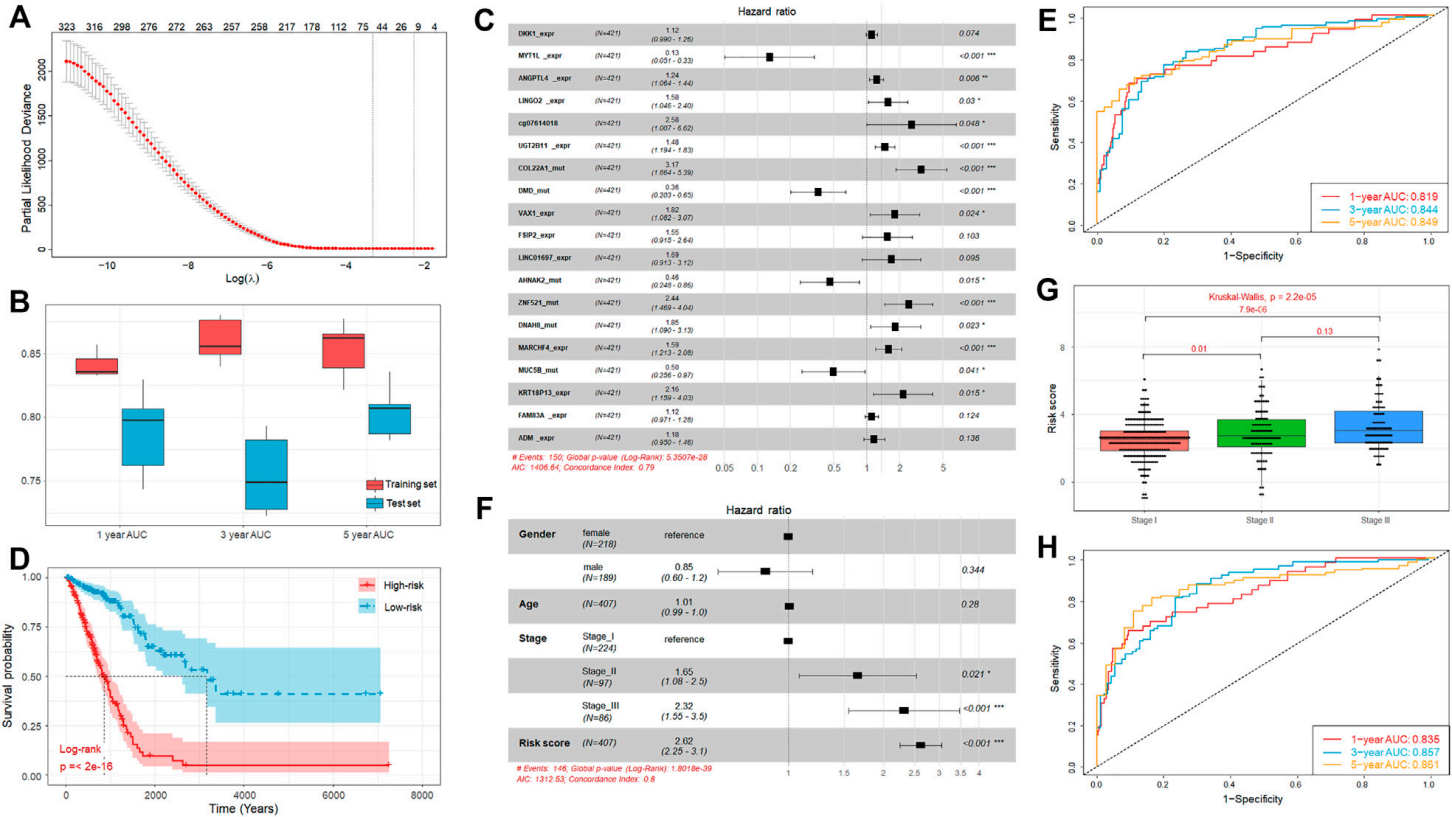
Establishment of prognostic model integrating multi-omics signatures. **(A)** Identification of the optimal penalization coefficient lambda in the Lasso regression model. **(B)** Boxplot of the 1-, 3-, and 5-year AUC values of the prognostic model established using multi-omics signatures in 5 repeated cross validations. **(C)** Forest plot of the prognostic impact of 19 genetic signatures. **(D)** Kaplan-Meier curves show the independent relevance between overall survival time and risk score. **(E)** ROC curves of the risk score for predicting 1-year, 3-year, and 5-year survival. **(F)** Forest plot of the prognostic impact of risk score and clinical factors. **(G)** Comparison of risk score of patients in Stage I, Stage II, and Stage III. **(H)** ROC curves of the risk score combined with clinical stage for predicting 1-year, 3-year, and 5-year survival.

To obtain a more comprehensive and robust model for the prognostic prediction, we integrated the multi-omics genetic signatures obtained above. At the transcriptome level, a total of 1,091 up-regulated and 1,707 down-regulated genes were identified in the “HYPOXIA_H & IMMUNITY_L” cohort. At the genome level, 181 and 44 frequently mutated genes were identified in the “HYPOXIA_H & IMMUNITY_L” and “HYPOXIA_H & IMMUNITY_L” cohorts, respectively. At the DNA methylation level, 1,163 out of 2,208 DMPs locating at the region of 645 annotated genes were differentially methylated between the “HYPOXIA_H & IMMUNITY_L” and “HYPOXIA_H & IMMUNITY_L” cohorts. Furthermore, we refined the hypoxia-immune-related prognostic signatures with significant effect on the overall survival time of patients from these genetic alterations based on univariate Cox proportional hazards model. After that, 336 items composed of 230 DEGs, 9 mutations, and 97 DMPs were selected. Considering the large number of significant signatures and possible interaction among them, we applied LASSO Cox regression model to evaluate the extent to which signatures contributes to predicting survival. Under the optimal parameter ln(λ) = −3.3 (Figure 5A), we reserved 39 signatures (27 DEGs, 8 mutations, and 4 DMPs) to establish the multivariate Cox proportional hazards regression model with stepwise method.

For the lack of the matching multi-omics data from other sources, we randomly divided TCGA samples into a training (70%, *n* = 295) and independent test set (30%, *n* = 126), and this process was repeated 5 times. The results showed that the performance of the trained models is satisfied with the average concordance index (C-index) equal to 0.816. Next, the risk score for each sample was calculated based on the established models, which has great discriminative power on survival status. The average AUC values of 1-, 3-, and 5-year prognosis prediction on training sets reached 0.841, 0.86, and 0.853 (Figure 5B). With regard to the prediction on the test sets, the performance exhibited a similar performance with the average AUC value of 1-, 3-, and 5-year survival equal to 0.788, 0.755, and 0.805 (Figure 5B). Moreover, the samples were classified into high-risk and low-risk cohorts by median-risk score. Kaplan-Meier survival analysis showed that the high-risk cohorts had a poorer overall survival compared with the low-risk cohort (*p*-value < 0.001, Supplementary Figure S3).

The above results revealed the great robustness and validity of the strategy of model construction, and we further combined all TCGA samples and generated an overall prediction model comprised of 19 signatures, including 11 DEGs, 7 mutations, and 1 DMPs (Figure 5C), from which we found some signatures, such as DEGs FSIP2, LINC01697, FAM83A, and ADM, seemed to be statistically insignificant initially (*p*-value > 0.05) but are likely associated with other signatures and outcome. The contributions of these 19 signatures on the overall model are listed in Table 1.

**TABLE 1:** Nineteen signatures associated with overall survival by multivariate Cox regression analysis.

ID	Coefficient	HR	HR_95L	HR_95H	*p*-value
DKK1_expr	0.108858	1.115004	0.989575	1.25633	0.0738
MYT1L_mutation	−2.04102	0.129897	0.050676	0.332959	2.14E-05
ANGPTL4_expr	0.213808	1.238385	1.064096	1.441223	0.005732
LINGO2_expr	0.459297	1.582961	1.045753	2.396135	0.029895
cg07614018	0.948601	2.582095	1.007391	6.618301	0.048234
UGT2B11_expr	0.389956	1.476916	1.194457	1.82617	0.000317
COL22A1_mutation	1.153178	3.168244	1.863983	5.38512	2.04E-05
DMD_mutation	−1.01334	0.363003	0.203103	0.648791	0.000626
VAX1_expr	0.599708	1.821587	1.08203	3.066624	0.024032
FSIP2_expr	0.441211	1.554588	0.915153	2.640809	0.102679
LINC01697_expr	0.523275	1.687546	0.9127	3.120206	0.095184
AHNAK2_mutation	−0.77174	0.462206	0.248079	0.861156	0.015066
ZNF521_mutation	0.890135	2.435457	1.469187	4.037234	0.000557
DNAH8_mutation	0.613942	1.8477	1.089994	3.132124	0.022609
MARCHF4_expr	0.463818	1.590134	1.212916	2.084666	0.000788
MUC5B_mutation	−0.69462	0.499262	0.256239	0.972773	0.041243
KRT18P13_expr	0.77069	2.161258	1.158634	4.031502	0.015399
FAM83A_expr	0.109214	1.115401	0.970587	1.281821	0.123754
ADM_expr	0.163852	1.17804	0.949734	1.461228	0.13603

In brief, the mutation of MYT1L, DMD, AHNAK2, and MUC5B have significantly positive contribution to a better prognosis, while the others played opposite roles. Moreover, similar to our above observation, the overall survival time of the high-risk cohort is significantly shorter than that of low-risk cohort (Figure 5D). Besides that, we also observed that high discriminative power of risk score for the 1-year, 3-year, and 5-year survival rates, according to the respective AUC value, were 0.819, 0.844, and 0.849 (Figure 5E). To further prove that integrating multi-omic characteristics can provide more robust prognostic prediction than using single-omic characteristics, we adopted the same strategy as mentioned above for each type of omic data. The results showed that no single-omic characteristics can provide a stronger model than the integrated model (Supplementary Figure S4).

In addition to the genetic alteration, we also consider some clinical factors which may also have prognosis ability, such as stage, gender, and age. We found that the clinical stage was significantly associated with the overall survival time, but the gender and age were not (Figure 5F). We tested the association between different clinical factors and the risk score, and we found that the risk scores of patients in Stage III and Stage II were significantly larger than those of patients in Stage I (Figure 5G). Combining these clinical factors with risk score, we established an incorporated model and the results showed that the prognosis ability can be improved through incorporating the risk score with stage information (C-index = 0.803). Besides that, the incorporated model can also achieve a better performance on its 1-year (AUC = 0.835), 3-year (AUC = 0.857), and 5-year (AUC = 0.861) survival predictions (Figure 5H). Hence, the multi-omics signatures comprising the above 19 genetic alteration can produce an accurate prognostic prediction and the risk score calculated based on these multi-omics signatures can be regarded as an independent prognostic indicator.

## Methods and Materials

### Patient Cohorts and Data Preparation

The Cancer Genome Atlas (TCGA) cohort consisted of 510 LUAD primary solid tumor samples and 58 normal control samples of RNA-seq profiles, 561 LUAD samples of WES data, and 455 profiles of the Illumina 450 k DNA methylation array, downloaded from the UCSC Xena browser (http://xena.ucsc.edu) (Goldman et al., 2020). To eliminate the error caused by the quantitative mRNA abundance of FPKM in multiple samples, we convert FPKM to TPM for standardization (Li et al., 2010).

### Immunity Status Definition

The expression data was transformed into Transcripts per Million. The immune-related genes (IRGs) for 28 immune cell subpopulations were obtained from Charoentong et al. (2017) study. Using the normalized gene expression data, we calculated the enrichment score (ES) of the 28 immune cell types for each sample through Gene Set Variation Analysis (GSEA) (Hänzelmann et al., 2013). Using the ES profile, the LUAD samples of expression profiles were classified into two groups by hierarchical clustering, and survival analysis was performed according the groups. Patients with good prognosis were assigned to IMMUNITY_H group and the others to IMMUNITY_L group. The ESTIMATE algorithm was used to generate an immune score through estimating the proportion of different infiltrating stromal and immune cells. The estimated scores between the high-immunity and low-immunity cohorts were compared using the Mann–Whitney U-test. By mapping the sample IDs of the RNA-seq profiles, we also constructed immune-related cohorts for the LUAD samples of both the WES and DNA methylation profiles.

### Identification of Hypoxia-Immune-Related Subtypes

The hypoxia related genes (HRGs) were downloaded from the molecular marker database (MSigDB v7.4). To deduce the hypoxia status, we applied the algorithm of uniform manifold approximation and projection (UMAP), which can be used for general non-linear dimension reduction, to reduce the dimension of the HRG expression profile, and the latent variable was used to cluster the patients into two groups using hierarchical clustering with “ward.D” agglomeration method. The survival analysis was performance on both the groups and the patients with poorer prognosis were regarded as HYPOXIA_L group and the others as HYPOXIA_H. Combining the immune status, we further divided the patients into three groups, which were “HYPOXIA_L & IMMUNITY_H,” “HYPOXIA_L & IMMUNITY_H,” and “MIX” groups.

### Multi-Omics Data Analysis and Prognosis Prediction Model Construction

In this study, we aimed to investigate the difference in gene expression, somatic mutations, and DNA methylation between the “HYPOXIA_L & IMMUNITY_H” and “HYPOXIA_L & IMMUNITY_H” cohort, respectively. The differential gene expression analysis between the two cohorts were identified using DESeq2 package (Anders et al., 2013) with the RNA-seq raw count data. Genes with fold change larger than two and adjusted *p*-value less than 0.001 were regarded as significantly differentially expressed. For Somatic mutations, the SNVs, SNPs, and INDELs were detected using VarScan2 (Koboldt et al., 2012; Lu et al., 2019a), and the results were downloaded from the UCSC Xena browser. Fisher’s exact test was used to identify the differential mutation patterns, and genes with adjusted *p*-value less than 0.01 were regarded as differentially mutated genes. The co-occurrence and mutually exclusive mutations were identified using the CoMEt algorithm (Leiserson et al., 2015). For DNA methylation, we first filtered the samples with more than 20% missing values and the remaining miss values were imputed using “*champ.impute*” function in R package “ChAMP.” The differentially methylation probes were also identified using the “*champ.DMP*” function in R package “ChAMP.”

As the survival time and status were used to evaluated the prognosis of LUAD patients, we only used cases with an overall survival record to construct the prognosis prediction model based on gene expression, DNA methylation, and somatic mutation data, respectively. For the multi-omic integration-based model, only the cases with all three types of omic-data were used. The univariate Cox proportional hazards regression was first used to assess the individual effect of every alteration and features with *p*-value less than 0.05 were retained. After that, we used the lasso regression method to further filter the less informative features. At last, the multivariate Cox proportion hazards regression with a stepwise procedure was used to reduce the redundant variables, and formed the final prognosis prediction model. The risk score for each sample was calculated based on the coefficients provided by the model. The C-index which was used to evaluated the performance of the prognosis prediction model was calculated using R package “survival.” The 1-, 3-, and 5-year receiver operating characteristic (ROC) curve were generated using the R package “timeROC.”

### Functional Enrichment Analysis

GO analysis was performed as our previous studies (Lu et al., 2019b; Lu et al., 2019c; Lu et al., 2019d). The analysis of DEG and DMP-associated genes and GSEA were performed using the R package “clusterProfile.” The GSEA plot was generated using the R package “enrichplot.”

## Discussion

Considering the heterogeneous outcomes and diverse therapeutic responses of LUAD, it is essential to establish a robust predictor to evaluate the risk and prognosis of patients. Immune and hypoxia were reported to play a critical role in the tumor initiation and progression, and significantly associated with prognosis. However, comprehensive analysis of genetic alterations related to both the immune and hypoxia remains far from satisfactory. In this study, we take into account the immune and hypoxia signal and perform a multi-omics integrative analysis.

Based on the expression profile of immune and hypoxia related genes, we defined the immune and hypoxia status for each patient, respectively. As expected, the immune status is positively associated with prognosis, while the hypoxia status is the opposite. Combining both the immune and hypoxia status, the patients were further divided into three cohorts, which are “HYPOXIA_H & IMMUNITY_L,” “HYPOXIA_H & IMMUNITY_L,” and “MIX”. After that, we investigated the genetic alterations between the first two cohorts from the gene expression, DNA methylation, and somatic mutation layers and gave the signatures accordingly. With multi-step strategy, we refined 19 signatures and established a robust model stratify patients with different risks and prognosis.

Although our study provided a more comprehensive landscape of the hypoxia-immune genome map, there are several limits that need deeper investigation. As our model contains three types of omic data, including RNA-seq, WES, and DNA methylation array data, we cannot find other datasets to validate our model, which is the main limitation of our study. Moreover, as we all know, LUAD is very complex and heterogenous, and it is difficult to cover all variations among the patients. Even so, with the rapid development of biological technologies, more researchers adopt multi-omics strategy to resolve the initiation and progression of LUAD. Besides that, our comprehensive characterization of genetic alteration from different omic layers between the “HYPOXIA_H & IMMUNITY_L” and “HYPOXIA_H & IMMUNITY_L” status can also be solely used. Moreover, our prognostic prediction model can potentially exhibit compelling clinical value that may lead to the improvement of overall survival and even for the development of new therapeutic strategies for LUAD patients.

## Data Availability

The datasets presented in this study can be found in online repositories. The names of the repository/repositories and accession number(s) can be found in the article/Supplementary Material.
