# Supplementary figures and images for: Multi-Omics Signatures Identification for LUAD Prognosis Prediction Model Based on the Integrative Analysis of Immune and Hypoxia Signals
Source: Front Cell Dev Biol. 2022 Mar 10;10:840466. doi: 10.3389/fcell.2022.840466 (PMC8960258; doi:10.3389/fcell.2022.840466)

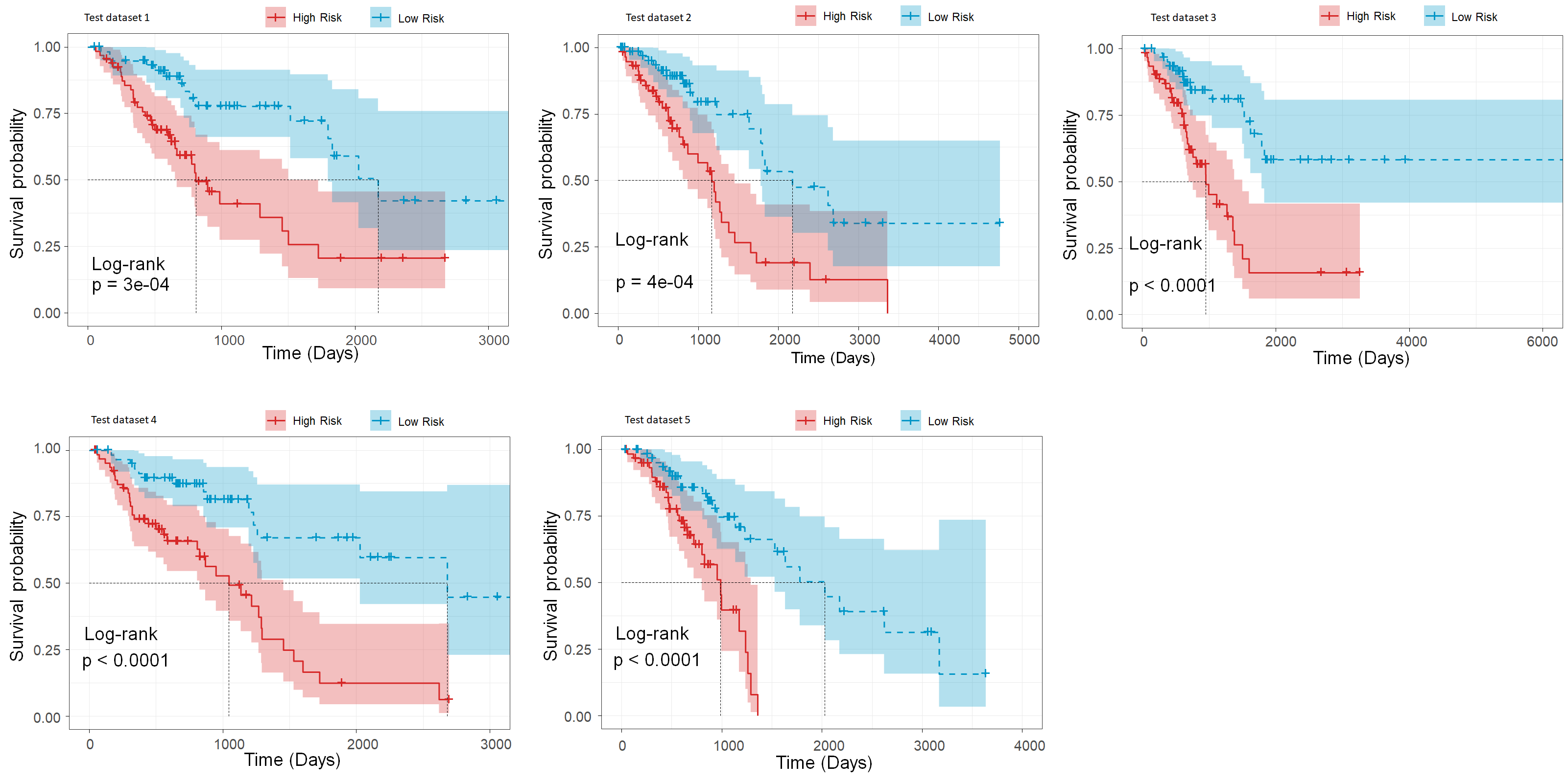

Supplement: Supplementary file 3 [file Image3.TIF]

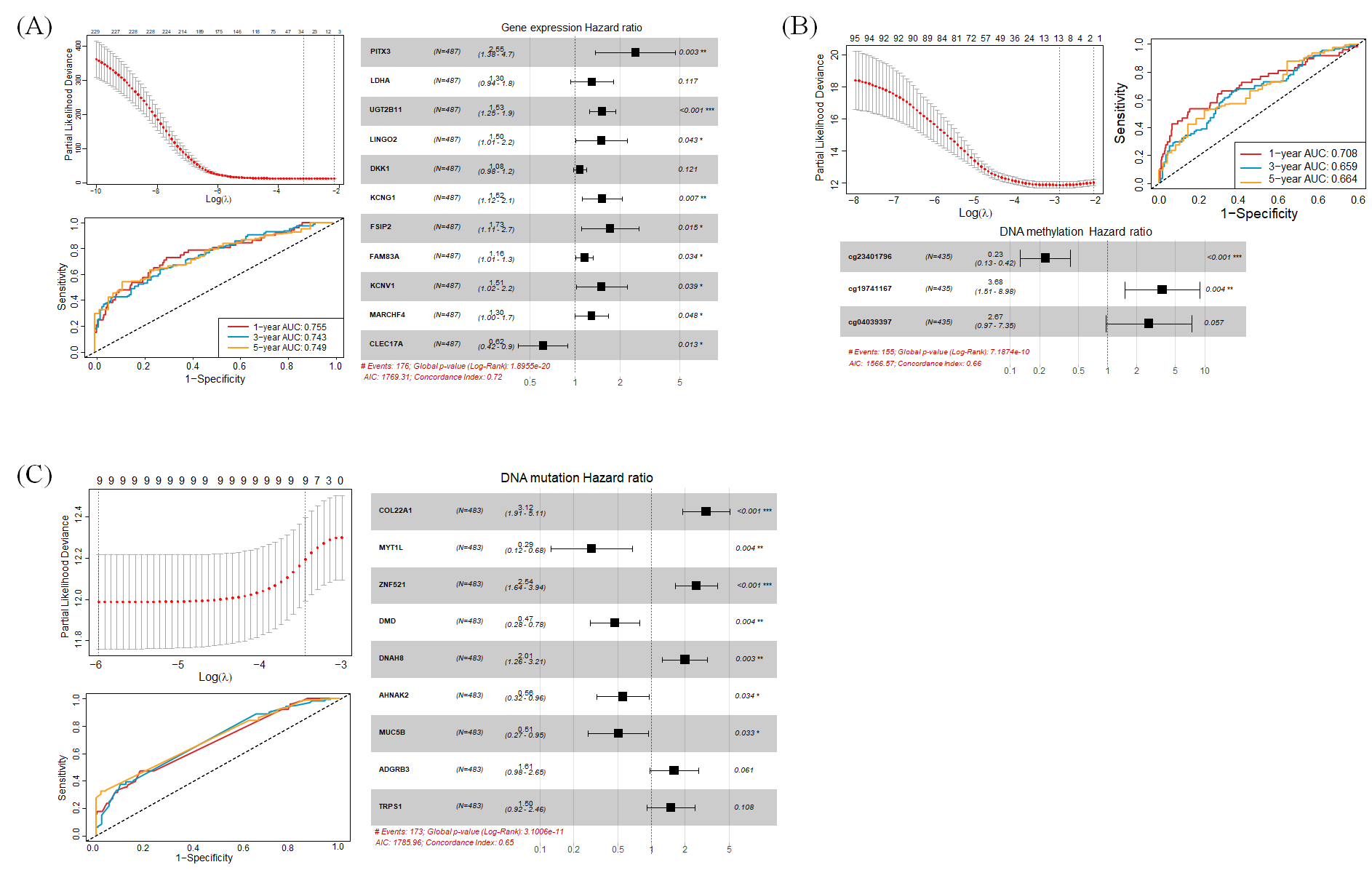

Supplement: Supplementary file 4 [file Image4.TIF]

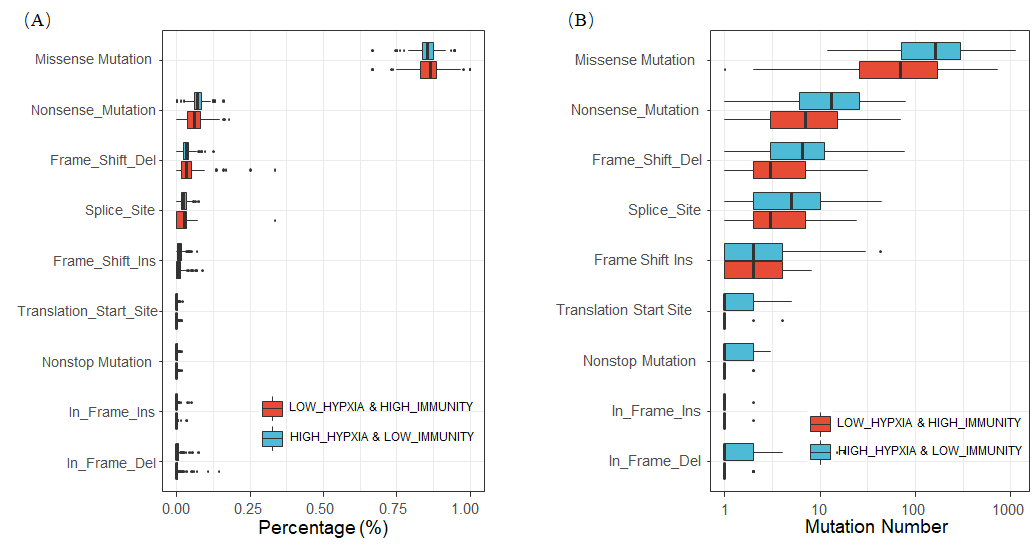

Supplement: Supplementary file 5 [file Image2.TIF]

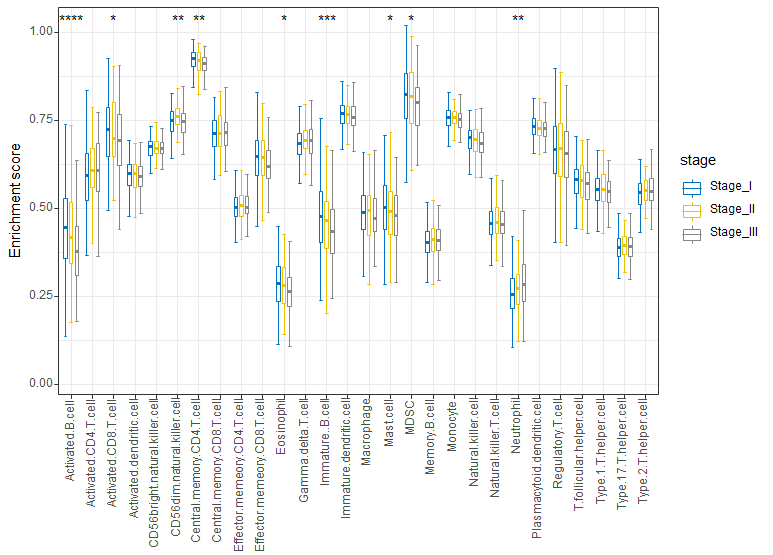

Supplement: Supplementary file 6 [file Image1.TIF]
